# A Wearable Fingertip Force Feedback Device System for Object Stiffness Sensing

**DOI:** 10.3390/mi15060693

**Published:** 2024-05-24

**Authors:** Changcheng Wu, Jianli Ren, Qingqing Cao, Zeran Yue, Ting Fang, Aiguo Song

**Affiliations:** 1 School of Instrument Science and Engineering, Southeast University, Nanjing 210096, China; a.g.song@seu.edu.cn; 2 College of Automation Engineering, Nanjing University of Aeronautics and Astronautics, Nanjing 211100, China; jianliren@nuaa.edu.cn (J.R.); yzr_de@163.com (Z.Y.); tingfang@nuaa.edu.cn (T.F.); 3 School of Aeronautic Engineering, Nanjing Vocational University of Industry Technology, Nanjing 210023, China; caoqingqing87@gmail.com

**Keywords:** wearable haptics, force feedback device, virtual reality, human-computer interaction

## Abstract

Virtual reality technology brings a new experience to human-computer interaction, while wearable force feedback devices can enhance the immersion of users in interaction. This paper proposes a wearable fingertip force feedback device that uses a tendon drive mechanism, with the aim of simulating the stiffness characteristics of objects within virtual scenes. The device adjusts the rotation angle of the torsion spring through a DC motor, and then uses a wire to convert the torque into a feedback force at the user’s index fingertips, with an output force of up to 4 N and a force change rate of up to 10 N/s. This paper introduces the mechanical structure and design process of the force feedback device, and conducts a mechanical analysis of the device to select the appropriate components. Physical and psychological experiments are conducted to comprehensively evaluate the device’s performance in conveying object stiffness information. The results show that the device can simulate different stiffness characteristics of objects, and users can distinguish objects with different stiffness characteristics well when wearing the force feedback device and interacting with the three-dimensional virtual environments.

## 1. Introduction

Virtual Reality (VR), as a method of human-computer interaction, can simulate the scenes and sounds in the environment to create an immersive experience. VR-based force feedback technology allows users to use tactile rendering algorithms to calculate the feedback force generated during the interaction [1,2,3]. These calculated forces are then transmitted to users through haptic feedback devices [4]. Users can also touch, perceive, and manipulate virtual objects using haptic feedback devices [5]. Over the past few decades, force feedback technology has been widely applied in various industries, including medical rehabilitation [6,7], remote sensing operations [8], and more.

To explore virtual environments, a large number of force feedback devices have been developed [9]. Different parts of the human body have varying spatial perception capabilities. Compared with other areas, fingers have higher spatial resolution and can transmit more spatial information [10]. As the hands are the most frequently used parts of the body for interacting with the external world, most research on force feedback devices has been focused on the hands. The TouchX developed by Geometry Company (San Francisco, CA, USA) represents a typical example of force feedback devices, capable of providing lower friction to achieve smoother tactile sensations. Many researchers in the field of force feedback utilize this device to study haptic rendering algorithms [11,12]. However, grounded force feedback devices like this mounted on a fixed surface cannot be easily moved during use, thereby restricting user flexibility [13]. Handheld force feedback devices are mostly used for interaction in 2D scenarios such as touch screens, where users cannot actively grasp or touch with their hands, thus limiting hand mobility [14,15].

In recent years, wearable force feedback devices have been widely used in VR interactions. These devices relocate the grounding base as close as possible to the feedback stimulation point, making them compact and easy to operate. The driving methods of force feedback equipment include electromagnetic, pneumatic, hydraulic and motor-based systems [16]. Lu et al. designed a non-contact two-dimensional haptic rendering system based on electromagnetic force control [17]. By adjusting the current of the two-dimensional controllable background electromagnet, electromagnetic forces in the x and y directions are generated on the permanent magnets worn on the fingertips. Zhang et al. designed a lightweight two-finger force feedback glove based on pneumatic-driven soft actuators, using pre-deformation of the soft actuator to achieve the driving capability and free space feeling on the back of the hand [18]. When simulating a constrained space, the fingertip force can reach up to 2.1 N. Wang et al. improved the glove into a three-finger force feedback glove, with a maximum reverse driving force of 0.48 N in free space and fingertip forces of up to 4.09 N in constrained space [19]. Zhang et al. proposed a two-point contact fingertip tactile force feedback device, which controls two push rod-end effectors through a hydraulic circuit to generate two feedback forces at two contact points under the user’s finger pad, with a local force of up to 8 N at each point [20]. Devices based on electromagnetic feedback consume more energy and are more sensitive to external magnetic fields and electromagnetic interference. Meanwhile, devices based on pneumatic feedback and hydraulic pressure have lower accuracy and generally slower response speeds [21]. Finger cot-type devices based on a motor drive usually have a movable platform below the fingertip, which is driven by the motor to provide feedback force at the fingertip [4,22]. Martinez-Hernandez et al. proposed a device with a cylinder structure below the fingertip [23]. The device was controlled by two servos to provide tactile feedback by moving up and down and sliding feedback by rotating left and right. The rotating spherical wearable fingertip device designed by Park et al. uses a small servo motor and a motor connected to an articulated leg to drive the movement of a platform under the fingertip to provide fingertip feedback force [24]. The maximum output feedback force of this device is 4.7 N. However, wearable finger cot-type force feedback devices install all driving motors on the finger cuffs, resulting in a heavy burden on the fingers and it is difficult to consider the impact of different people’s fingertip size differences on feedback force accuracy [25,26]. Based on tendon drive, Lee et al. placed the drive motor on the back of the hand, which can produce strong enough kinesthetic feedback [22]. Kuang et al. connected four servo motor drive modules on the back of the hand to articulated arms, which were then connected to the end effectors on the fingers [27]. By separating the motor drive modules from the end actuators on the fingers, the complexity of the end actuator device is reduced, and the pressure on the fingertips is minimized.

This paper proposes a wearable force feedback device designed based on tendon drive for interaction in three-dimensional virtual environments, featuring haptic feedback of normal forces. The proposed device allows users to explore the different stiffness properties of virtual objects by freely moving their hands within the interactive scene.

The rest paper is organized as follows. Section 2 introduces the wearable fingertip force feedback device from the aspects of mechanical structure and mechanics, along with the selection of key components. Section 3 describes the design of the measurement and control system of the device. Section 4 conducts performance testing of the device. Section 5 conducts stiffness evaluation experiments to assess the device’s ability to reproduce different stiffness characteristics of objects.

## 2. Wearable Fingertip Force Feedback Device

The application of force feedback devices can enhance the operational experience in virtual environments, making it more intuitive and natural. By introducing the perception of force, users can more accurately perceive the texture, hardness, and other characteristics of virtual objects. This section will provide a detailed description of the mechanical structure and operation process of the designed wearable fingertip force feedback device, along with theoretical analysis of the device, exploration of factors influencing the output force of the device, and finally the parameter design and selection of the key components.

### 2.1. Mechanical Design

**Figure 1:**
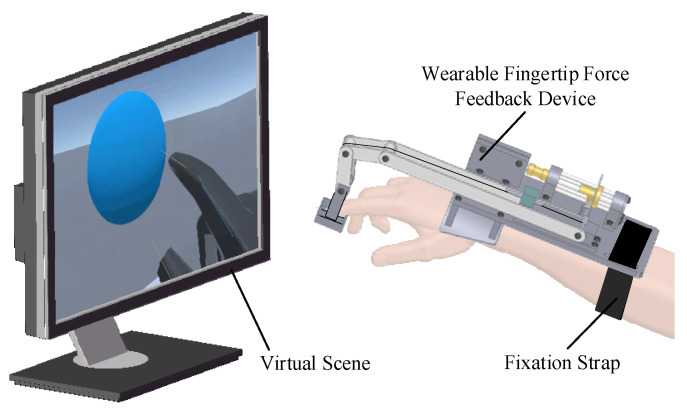
Wearable fingertip force feedback device interaction with virtual scene.

To alleviate the burden on the fingers, the device’s driving and control components are integrated onto a base attached to the back of the hand. Figure 1 shows the user interacting with the three-dimensional virtual environment while wearing the device, which is secured to the user’s hand with black fixed straps. The stiffness of objects are crucial for distinguishing between different object categories. When a finger actively presses an object, the feedback perception from different stiffness characteristics varies. The device is characterized by its ability to provide normal force to the index fingertip, simulating the stiffness of contact with virtual objects.

**Figure 2:**
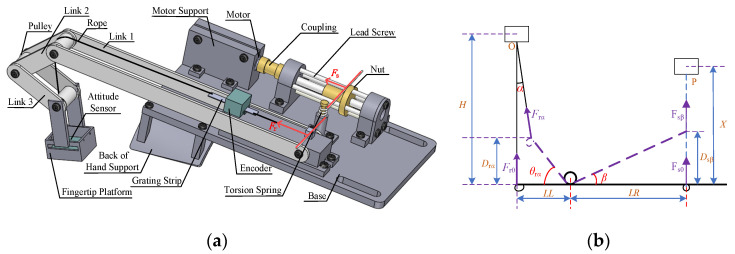
Fingertip force feedback device. (**a**) Mechanical details of the device. (**b**) The principle of device feedback force output.

Figure 2a shows the mechanical structure of the force feedback device. A torsion spring is a key component of the device. One arm of the torsion spring rests on the nut, and the other one is connected to the rope. The motor is connected to the screw through a coupling. When the control unit of the device receives the movement command, the motor drives the screw to rotate, causing the nut to move back and forth along the screw, thereby driving the torsion arm resting against the nut to rotate. As the relative angle between the two torsion arms changes, torque is generated. Due to the action of torque, the other arm of the torsion spring moves, lifting or lowering the height of the fingertip platform through the rope, providing feedback force to the user’s fingertip. There is an attitude sensor embedded in the fingertip platform, to measure the change in the fingertip’s attitude angle when the finger bends. The motor is installed in the motor support, and there are screws on the top of the support to hold the motor tightly, increase the friction between the support and the motor, and prevent the body from rotating when the motor is running. Link 1 is fixed on the link support, while link 2, link 3, and the fingertip platform can rotate freely. Pulleys are embedded between each connecting rod to reduce friction. The grating encoder is mounted on the connecting rod support and is used to measure the distance moved by a rope (grating strip) that passes through both ends of the grating strip.

The device is an independent unit, with its control unit containing a 12 V power supply and a Bluetooth communication module. To balance the accuracy and weight of the device, a combination of metal links, miniature lead screw nuts, and a resin-based base bracket is adopted in the paper. This design aims to minimize potential looseness caused by the 3D resin material, thereby enhancing the stability of force feedback.

### 2.2. Mechanical Analysis

To ensure the stability of the force feedback device during interactions, it is crucial to thoroughly understand the relationship between the tension applied to the fingertip platform by the traction rope and the distance moved by the nut.

In Figure 2a, the red lines indicate the forces acting on both ends of the torsion spring, which is the key position to achieve force feedback. A mathematical model is established in this area, and the force feedback performance of the device is analyzed using torsion springs as the research object, as shown in Figure 2b. In the following, the torsion arm that leans on the nut is referred to as the long torsion arm, and the end fixed to the rope as the short torsion arm.

In Figure 2b, *LL* is the length of the short torque arm of the torsion spring, and *LR* is the length of the long torque arm of the torsion spring. *θ_rα_* is the rotation angle of the short arm of the torsion spring relative to the horizontal direction, and *β* is the rotation angle of the long arm of the torsion spring relative to the horizontal direction. *α* is the deflection angle of the thin line in the vertical direction. In the initial position, *θ_rα_* = *β* = *α* = 0. *F_r_*_0_ is the force on the short torsion arm at the initial moment, that is, in the horizontal direction, and *F_rα_* is the force on the short torsion arm when the thin line deflection angle is *α*. *F*_s0_ is the force on the long torsion arm at the initial moment, that is, in the horizontal direction, and *F_sβ_* is the force acting on the long torsion arm when the angle is *β*. *D_rα_* is the vertical distance between the action point of the short torsion arm and the initial position, *D_sβ_* is the vertical distance between the action point of the long torsion arm and the initial position, *H* is the vertical distance between the short torsion arm in the initial position and the pulley on connecting link1, and *X* is the vertical distance between the long torque arm in the initial position and the bearing seat. The specific parameters of the device are shown in Table 1.

After the device has been worn, the link structure will adaptively adjust according to the position of the hand, without restricting the free movement of the user’s hand. When analyzing the stress on the index fingertip, the movement of the device can be divided into two situations: first, when the motor does not work, the user actively bends the finger, causing rotation of the short torsion arm; second, when the user does not bend the finger, the motor runs, causing displacement of the nut, and rotation of the long torsion arm.

In the first case, it is assumed that the nut and the long torsion arm are in the initial position (*D_sβ_* = *β* = 0). When the finger actively bends, the index fingertip will press the platform, and the platform then pulls the rope, and the rope pulls the short arm of the torsion spring, causing *θ_r_* to rotate to the angle of *θ_rα_*. At this point, the magnitude of the force is *F_rα_* and the deflection angle of the force is *α*. Based on the moment balance equation and the torque formula of the torsion spring, the following equation is established:

(1) $$F_{r\alpha }\cos\alpha \times LL\cos\theta_{r\alpha }-F_{r\alpha }\sin\alpha \times D_{r\alpha }=k_{s}\theta_{r\alpha }\frac{180}{\pi },$$

where *k_s_* is the stiffness coefficient of the torsion spring. Since *θ_rα_* is in radians while the torsion spring formula typically requires degrees, conversion is necessary. From *D_rα_* = *LL*sin*θ_rα_*, after rearranging (1), the force at the index fingertip can be obtained as:

(2) $$F_{r\alpha }=\frac{k_{s}\theta_{r\alpha }\frac{180}{\pi }}{LL\cos(\theta_{r\alpha }+\alpha )},$$

From Figure 2b, it can be observed that there is the following geometric relationship between *θ_rα_* and *α*:

(3) $$\alpha =\arctan(\frac{LL(1-\cos\theta_{r\alpha })}{H-LL\sin\theta_{r\alpha }}),$$

In the second case, it is assumed that the current short torsion arm is in the initial position *D_rα_* = *θ_rα_* = 0. When the motor is working, the position of the nut will change. Assume that the nut advances a distance *D_sβ_* from the initial position, the rotation angle of the long torsion arm is *β*, and the force on the long torsion arm is *F_sβ_*. In this case, based on torque equilibrium equation and the torsional torque of the torsion spring, the following equation can be established:

(4) $$F_{r0}\cdot LL=k_{s}\beta \frac{180}{\pi }=k_{s}\arctan(\frac{D_{s\beta }}{LR})\frac{180}{\pi },$$

The force at the index fingertip can be expressed as:

(5) $$F_{r\alpha }=F_{r0}=\frac{k_{s}}{LL}\arctan(\frac{D_{s\beta }}{LR})\frac{180}{\pi },$$

After combining the above two situations, the feedback force of the index fingertip calculated by combining (2) and (5) is as follows:

(6) $$F_{r\alpha }=\frac{k_{s}(\arctan(\frac{D_{s\beta }}{LR})+\theta_{r\alpha })}{LL\cos(\theta_{r\alpha }+\arctan(\frac{LL(1-\cos\theta_{r\alpha })}{H-LL\sin\theta_{r\alpha }}))}\frac{180}{\pi },$$

It can be seen from the above formula that the feedback force output at the index fingertip is related to the displacement *D_sβ_* of the adjusting nut and the rotation angle *θ_rα_* of the short torsion arm. Among them, *D_sβ_* can be adjusted by position control of the motor, and *θ_rα_* can be known by the change in the length of the rope Δ*s*. According to Figure 2b, there is the following geometric relationship:

(7) $$\Delta s=H-\frac{H-D_{r\alpha }}{\cos\alpha }=H-\frac{H-LL\sin\theta_{r\alpha }}{\cos\alpha },$$

It can be seen from (7) that *θ_rα_* and Δ*s* cannot be separated directly. Considering the approximation method, since the *α* value is very small, the maximum value is arctan *(LL*/*N*), which is 7.125°. Therefore, by cos(*α*) ≈ 1, the following approximate formula is obtained:

(8) $$\theta_{r\alpha }=\arcsin(\frac{H-(H-\Delta s)\cos\alpha }{LL})\approx \arcsin(\frac{\Delta s}{LL}),$$

In summary, when it is necessary to provide a feedback force of size *F_rα_* to the index fingertip, if the current finger bending stroke Δ*s* is known, force feedback can be achieved by controlling the motor to move the nut to the position *D_sβ_*, as shown in the formula:

(9) $$D_{s\beta }=LR\cdot \tan(-\theta_{r\alpha }+F_{r\alpha }\cdot \frac{\pi }{180}\cdot \frac{LL\cos(\theta_{r\alpha }+\arctan(\frac{LL(1-\cos\theta_{r\alpha })}{H-LL\sin\theta_{r\alpha }}))}{k_{s}}),$$

In particular, when the finger does not actively bend, that is, the finger’s stroke Δ*s* = 0, (9) can be simplified to:

(10) $$D_{s\beta }=LR\cdot \tan(F_{r\alpha }\cdot \frac{\pi }{180}\cdot \frac{LL}{k_{s}}),$$

### 2.3. Part Selection

According to the analysis of the force feedback device in the previous section, it is known that the parameters affecting the feedback force include the change in the length of the rope Δ*s*, the displacement of the nut *D_sβ_*, and the stiffness coefficient of the torsion spring *k_s_*. Among them, the displacement of the nut is controlled by the motor and the screw rod, so the rotation speed of the motor and the pitch of the screw rod will indirectly affect the performance of the force feedback. In this section, according to the performance requirements of the force feedback device, the motor, the pitch of the screw and the stiffness coefficient of the torsion spring are reasonably designed.

In order to ensure that the user has enough free space to move after wearing the device, the range of the cord’s stroke after the finger is bent and lifted is set to 0~10 mm, that is, 0 < Δ*s* < 10 mm. The maximum output feedback force *F_rα_* is 4 N. Substituting Δ*s* = 10 mm into (8), the maximum value of *θ_rα_* is 0.41 rad. Based on (3), the corresponding *α* is 0.01 rad. According to (2), *k_s_* should satisfy the following relationship:

(11) $$k_{s}\geq \frac{F_{r\alpha }LL\cos(\theta_{r\alpha }+\alpha )}{\theta_{r\alpha }}\frac{\pi }{180}\Rightarrow k_{s}\geq 3.887,$$

Since the required change rate of the feedback force of the device is greater than 10 N/s. From (5), the rate of change in force can be obtained as:

(12) $$\frac{dF_{r\alpha }}{dt}=\frac{k_{s}}{LL}\arctan(\frac{dD_{s\beta }}{dt}\cdot \frac{1}{LR})\frac{180}{\pi },$$

The relationship between the movement rate of the nut, the speed of the motor and the pitch of the screw is as follows:

(13) $$\frac{dD_{s\beta }}{dt}=\frac{n\cdot p}{60},$$

where the left side of the equation is the movement rate of the nut, *n* is the rotation speed of the motor in revolutions per minute (RPM), and *p* is the pitch of the screw rod in mm. Substituting the minimum value of *k_s_* is 3.887 obtained from (11) and the relationship of (13) into (12), the following inequality relationship is obtained:

(14) $$n\cdot p\geq 60\tan(\frac{12\times \frac{\pi }{180}\times LL}{k_{s}})/LR,$$

After calculating the product of the motor’s rotational speed and the lead of the screw is *n*·*p* ≥ 3348.7. Considering the spatial layout and volume dimensions of the device, it is preferable to choose a motor with a small form factor and high speed. For this device, the RE10 series DC motor manufactured by Maxon is chosen, with a rotational speed of 780 rpm, and paired with a screw with a lead of 6 mm. This yields *n*·*p* ≥ 4680, and the rate of change in the fingertip force under this set of parameters can exceed the preset conditions.

In addition, the torque of the motor needs to be able to meet the torque required for nut movement and should satisfy the following relationship: *F_motor_* ≥ *F_sβ-max_*. Here, *F_motor_* is the thrust of the motor, and *F_sβ-max_* is the maximum force of the long torque arm. The thrust of the motor can be expressed as:

(15) $$F_{motor}=\frac{2\pi P}{p\cdot n},$$

where, *P* is the power of the motor, *n* is the rotation speed of the motor, and *p* is the pitch of the screw. The maximum force on the long torsion arm can be expressed by the following formula:

(16) $$F_{s\beta -\max}=\frac{ks(\theta_{r\alpha -\max}+\beta_{\max})}{LR},$$

where, *θ_rα-max_* is the maximum value of *θ_rα_* and *β_max_* is the maximum value of *β*. According to the maximum value and (8), *θ_rα-max_* = 0.411 rad can be obtained. The maximum displacement of the nut *D_sβ_* is 20 mm, so the maximum rotation angle of the long torsion arm is 0.588 rad.

By substituting (15) and (16) into (14), we obtain the following inequality relationship:

(17) $$k_{s}\leq \frac{2\pi \cdot 9550\cdot P}{p\cdot n}\cdot \frac{LR}{\left(\theta_{r\alpha }+\beta \right)}\Leftrightarrow k_{s}\leq 10.08,$$

Combining (11) and (17), the stiffness coefficient range of the torsion spring can be obtained as 3.887 ≤ *k_s_* ≤ 10.08. The stiffness coefficient of the torsion spring can be expressed by the following formula:

(18) $$k_{s}=\frac{E\cdot d^{4}}{1167\cdot D_{m}\cdot \pi \cdot n},$$

where *E* is the elastic modulus of the torsion spring, *d* is the wire diameter of the torsion spring, *D_m_* is the middle diameter of the torsion spring, and *n* is the number of turns of the torsion spring. This article selected a torsion spring whose material is carbon steel, elastic modulus *E* = 194,000 MPa, wire diameter *d* = 1.2 mm, middle diameter *D_m_* = 5.0 mm, and number of turns *n* = 3. After calculation, its stiffness coefficient *k_s_* is 6.1.

**Figure 3:**
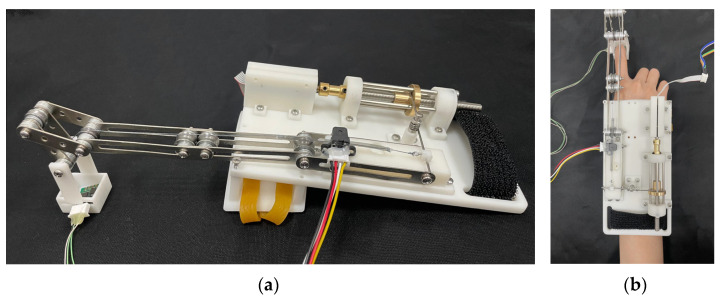
The picture of the device. (**a**) Wearable fingertip force feedback device prototype. (**b**) Device worn on human hand.

According to the device’s performance requirements, the parameter design and component selection for the motor speed, screw pitch, and torsion spring stiffness coefficient are completed. The depiction of the device is illustrated in Figure 3.

## 3. Measurement and Control System

The device is designed for interacting with objects in a three-dimensional (3D) environment, requiring the mapping of the user’s hand position to the 3D scene and displaying a virtual hand in the virtual environment to guide the user’s positioning. In virtual reality (VR), Leap Motion (UltraHaptics, Bristol, UK) visual sensors are commonly used to capture hand and finger movements. However, Leap Motion has problems with low accuracy and poor stability, and its accuracy is usually greater than 10 mm. Additionally, due to occlusion caused by wearing the device on the hand, Leap Motion often struggles to accurately detect finger joint information compared to palm areas, leading to potential misinterpretations. The low accuracy and instability can also affect the calculation of feedback forces, thus diminishing user experience. Considering these problems, a single Leap Motion may not be able to accurately detect the movement of the user’s fingers. Therefore, a multi-sensor fusion approach is adopted to control the position of the virtual hand. This paper utilizes Leap Motion for positioning the palm while employing both a grating encoder and attitude sensor for precise positioning of the index fingertip relative to the palm.

**Figure 4:**
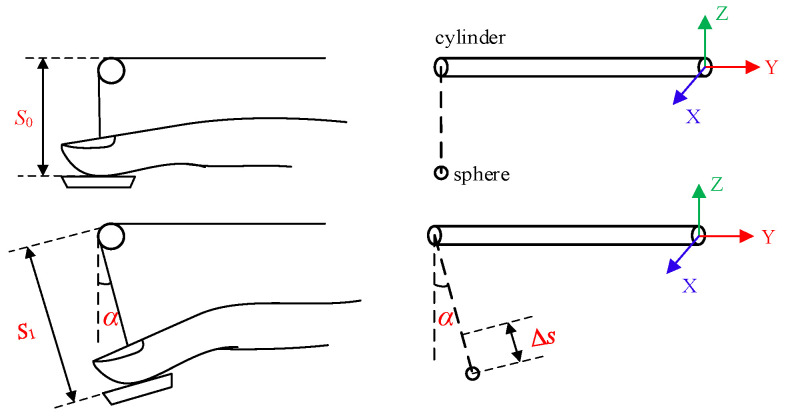
Simplified model of finger movement.

In the virtual scene, the virtual hand model is replaced with a simple geometry, and the motion control of the entire hand is simplified to the motion control of a single finger. The schematic diagram is shown in Figure 4. The sphere represents the contact point between the index fingertip and the fingertip platform of the force feedback device. The cylinder represents the connecting rod of the force feedback device, while the XY plane where the cylinder is located represents the palm. Assume that in the initial position, the distance between the left end points of the sphere (fingertip) and the cylinder (connecting rod) is *S*0. As the finger bends, the distance increases to *S*1. The finger’s stroke is Δ*s* = *S*1 − *S*0, which can be measured by a grating encoder; assuming that the movement of the finger is limited to the YZ plane, in the initial position, the deflection angle of the sphere (fingertip) relative to the Z axis is 0, and as the finger bends, the deflection angle becomes α, that is, the attitude angle of the index fingertip on the Z-axis is α, which can be measured by the attitude sensor.

**Figure 5:**
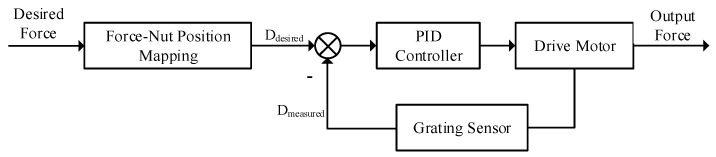
Output force control strategy.

The movement of the hand affects the change in the position of the rope. At the same time, the short arm of the torsion spring is also be pulled to rotate at a certain angle. After the theoretical calculation in Section 2, it is known that to output a specified feedback force at the fingertip, the moving position of the nut is related to the rotation angle of the short arm of the torsion spring. Therefore, the control method shown in Figure 5 is adopted in the motor driving part.

**Figure 6:**
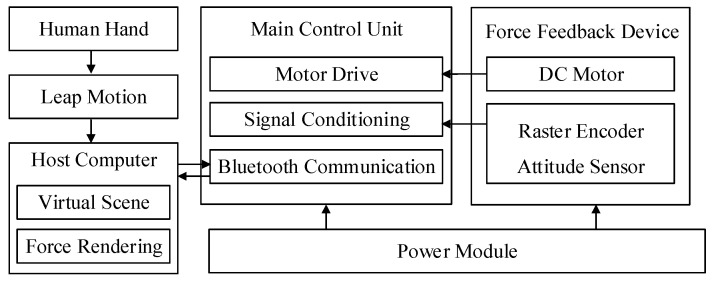
Schematic diagram of the measurement and control system of the device.

Figure 6 shows the schematic of the measurement and control system. The data detected by the attitude sensor is wirelessly transmitted to the host computer via Bluetooth. The host computer combines the data of Leap Motion to obtain the position mapping of the finger in the three-dimensional scene, and calculates the fingertip feedback force that should be output at the contact point. Subsequently, the host computer sends instructions to the drive board of the force feedback device through Bluetooth wireless transmission to drive the motor, thereby outputting feedback force at the index fingertip.

## 4. Device Performance Evaluation

In this section, static and dynamic test experiments of the device are conducted to evaluate the performance and ensure the reliability of the device in interacting with the virtual environment. Evaluate the accuracy, consistency and repeatability of the device through static experiments, and the dynamic experiment is used to detect the feedback force output response speed of the device.

### 4.1. Static Performance Test Experiment

**Figure 7:**
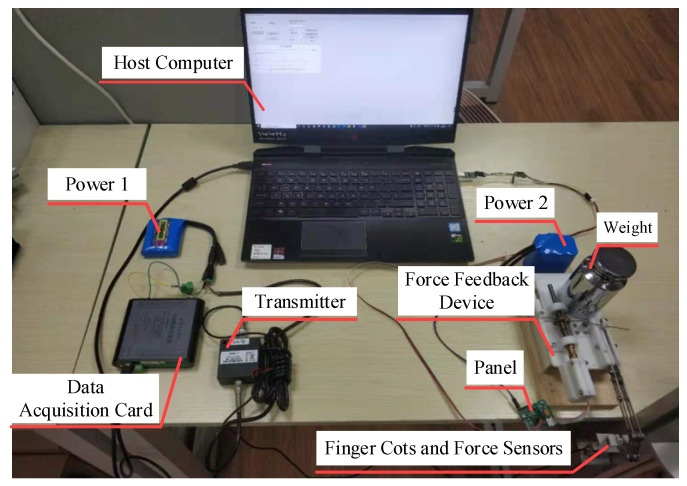
Fingertip force feedback device performance evaluation of the experimental platform.

Figure 7 shows the experimental scene of the device performance evaluation experiment, including the host computer, power supply, fingertip force feedback device, force sensor (ZNHM-VI-10Kg, Bengbu Zhongnuo Company, Bengbu, China), transmitter (ZNBSQ-V, Bengbu Zhongnuo Company, Bengbu, China) and data acquisition card (USB5936, Altai Technology Company, Beijing, China). The base of the force feedback device is fixed on a horizontal desktop, with the rear end secured by weights to prevent the device from tipping forward after being subjected to force at the fingertip platform. The tension sensor is connected to the bottom of the finger-end platform, which measures the tensile force exerted by the finger-end platform in the vertical direction, and the measured results are sent to the host computer for subsequent processing. The host computer software (Qt Creator 4.11.1) sends the target value of the feedback force to the control unit of the device. After receiving the command, the control unit drives the motor to generate upward pulling force on the fingertip platform. At the same time, while the force sensor measures the actual output pulling force.

**Figure 8:**
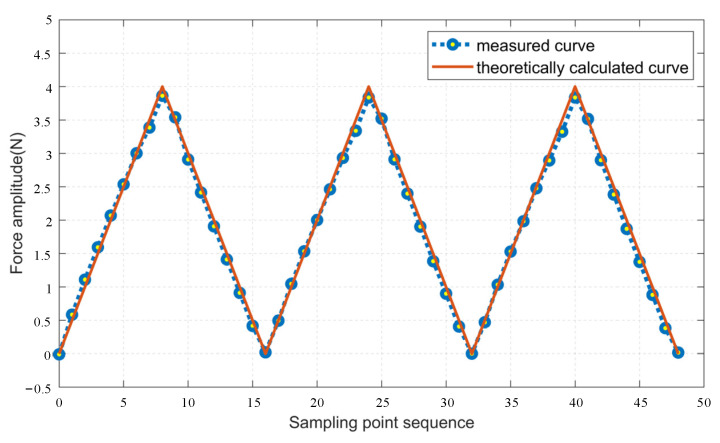
Comparison of measured and theoretically calculated output force.

Starting from 0 N, the computer sends the target value of the feedback force to the device, increasing by 0.5 N each time until it reaches 4 N, and then decreasing by 0.5 N each time until it returns to 0 N. Three sets of experiments are conducted, and the experimental results are shown in Figure 8.

In order to evaluate the device’s reproducibility of feedback force, accuracy, consistency, and repeatability are calculated and analyzed using three sets of measured experimental data.

(1) Accuracy

Accuracy is a critical indicator used to measure the accuracy of the device’s output feedback force. It is expressed by calculating the error between the measured force amplitude and the theoretically calculated force amplitude at each sampling point and taking the average value. The calculation formula is as follows:

(19) $$P=\sqrt{\frac{\sum_{i=1}^{n}{\left(y_{i}-y\right)}^{2}}{n}},$$

where *P* is the accuracy, *n* represents the number of sampling points, *y_i_* represents the measured force amplitude of the *i*th sampling point, and *y* represents the theoretically calculated force amplitude. By substituting the data from the three sets of experiments into the calculation, the average error obtained is 0.0755 N, and the maximum error is 0.23 N.

(2) Consistency

Consistency refers to the degree of overlap between the output signals when the input signals of the device are in the forward and reverse strokes. The maximum deviation of the output signal between the forward stroke and the reverse stroke is called the maximum hysteresis error. The ratio between the maximum hysteresis error and the full-scale output value, known as the maximum hysteresis rate, is usually used to express the relative error. The formula is as follows:

(20) $$E_{\max}=\frac{\max\left(\left|y_{i}-y_{d}\right|\right)}{Y_{FS}}\times 100\%,$$

where, *y_i_* is the amplitude of the output signal during the forward stroke, *y_d_* is the amplitude of the output signal during the reverse stroke, and *Y_FS_* is the maximum output value. After calculation, the maximum hysteresis error of the device is 0.198 N, and the maximum hysteresis rate is 4.95%.

(3) Repeatability

Repeatability refers to the degree of overlap of the output characteristic curves of the device when the output signal operates at full scale in the same direction. The calculation formula of repeatability error *E* is:

(21) $$E=\pm \frac{\left(2\sim 3\right)\overset{\wedge }{\sigma }}{Y}\times 100\%,$$

where, $\hat{\sigma }$ is the standard meter deviation, *Y* is the output value of the full scale, and the calculation formula of $\hat{\sigma }$ is as follows:

(22) $$\hat{\sigma }=\sqrt{\frac{\sum_{i=1}^{n}{\left(y_{i}-\bar{y}\right)}^{2}}{n-1}},$$

where, *y_i_* is the measured value of the *i*th sampling point, and $\bar{y}$ is the average value of each measured value. After calculation, the repeatability error is 1.9125%.

### 4.2. Dynamic Performance Test Experiment

**Figure 9:**
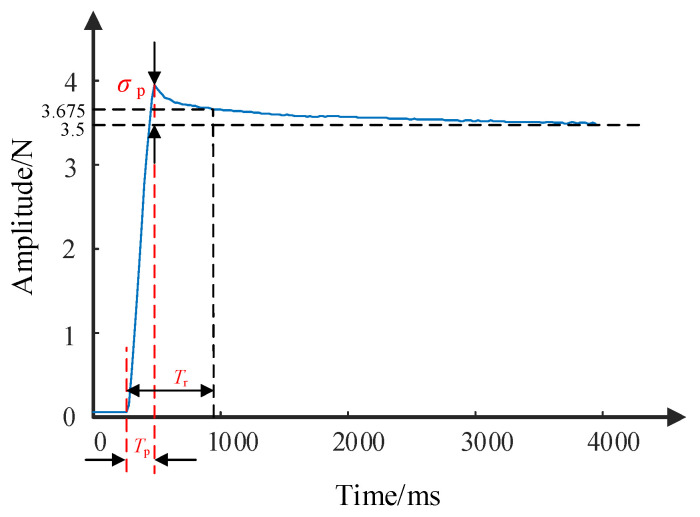
Step signal response curve with a peak value of 3.5 N.

The response time of the device refers to the time taken from issuing a command to completing the command content. In haptic feedback technology, the device’s fast response can improve the user interaction experience and efficiency. To assess the response speed of the device designed in this paper, a dynamic performance test experiment is carried out to measure the maximum overshoot *σ_p_*, peak time *T_p_* and response time *T_r_* of the device’s output feedback force. A series of eight step signals ranging from 0.5 to 4.0 N in peak value with 0.5 N intervals are used as input signals. Figure 9 illustrates the response curve of the step signal with a peak value of 3.5 N, and Table 2 presents the response characteristic parameters of each step signal.

According to the data in Table 2, it is observed that the overshoot of the output signal remains relatively consistent for any peak value of the input signal. The peak time range of the signal is 80~220 ms, with smaller amplitudes resulting in shorter peak times. For a step signal with a peak value of 1 N, the output peak time of the device is 100 ms, indicating that its response speed is greater than 10 N/s. While the response speed decreases for input step signals with a peak value of 0.5 N, the peak time remains below 100 ms. Therefore, during the interaction process, the device is capable of executing commands with peak values below 1 N within 100 ms. According to [28,29], for unexpected signals, the haptic interactive device should complete the output of fingertip force within 1 s after generating feedback force information. The response speed of the proposed device is greater than 10 N/s, which means if the maximum force feedback is less than 10 N during interactions with virtual environments while wearing the device, it can ensure a satisfactory user experience.

## 5. Object Stiffness Reproduction Experiment

In order to verify the effectiveness of the fingertip force feedback device in helping users perceive objects, during this research, a stiffness rendering interactive experiment was performed.

### 5.1. Experimental Method

**Figure 10:**
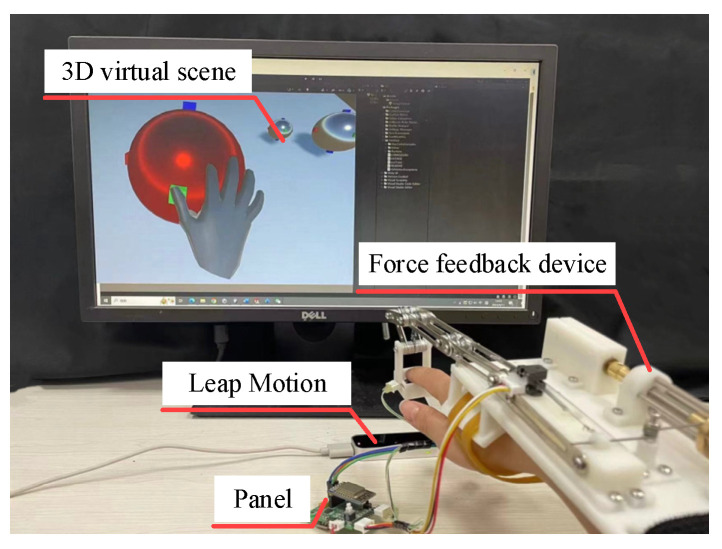
Experimental setup: virtual scene and interactive platform for object stiffness rendering tasks.

The virtual scene and interactive scene of the experiment are shown in Figure 10. The 3D virtual scene is built on the Unity platform, and a fusion method of Leap Motion and attitude sensor data are employed to determine whether the virtual hand’s fingertip are in contact with objects, with a collision detection module added to the fingertip. Taking into account the computational complexity and the model characteristics of the fingertip, a spherical bounding box algorithm is used to detect collisions. The collision results are visualized. When the color of the object is blue, it means that the user’s index fingertip is not touching the object. Conversely, when the color of the object is red, the situation is opposite. After the virtual hand’s index fingertip make contact with an object, it can continue to penetrate the object to simulate the process where the fingers compress the object in real-life situations. The object is deformed by force and reacts on the fingertip. Moreover, as the virtual fingertip penetrate deeper into the object, the feedback force on the fingertip increases.

In the stiffness perception experiment, three kinds of small balls with different stiffness are selected as experimental interaction objects. Except for the different stiffness, each small ball has the same shape and quality. The stiffness of these three balls is 0.5 N/mm, 1.0 N/mm, 1.5 N/mm, representing soft, medium and hard, respectively. The spring model based on Hooke’s law completes the corresponding force rendering on the surface of the virtual object. The equation is shown in the formula:

(23) $$F_{v}=k\cdot (r-d),$$

where, *k* is the stiffness of the ball, *r* is the radius of the ball, *d* is the distance between the lower surface of the fingertip and the center of the ball, and *F_v_* is the force on the virtual fingertip. By substituting the calculated feedback force into Formula (9), the distance *D_sβ_* where the nut needs to move can be obtained. The host computer sends corresponding commands to the control panel of the device, and drives the motor to rotate the nut to the corresponding position, so that the force on the real fingertip is the same as the force calculated on the virtual fingertip.

### 5.2. Experiment Procedure

Eight students (four male and four female) participate in this experiment, and all subjects are aged between 22 and 28 years old. The subjects have never worn this device before and are unaware of the purpose of the experiment. Before the formal experiment, the subjects are required to feel in advance the magnitude of the fingertip feedback force generated by the spheres of each stiffness. After the subjects became familiar with the entire operating environment, they are allowed to rest for 5 min to ensure that muscle memory in the testing environment would not affect the results of the formal experiment.

In order to prevent the noise from the operation of the motor in the device from interfering with perception, the subjects are required to wear headphones and play white noise during the entire experiment. During the experiment, a sphere with a stiffness characteristic will randomly appear in the virtual scene. The subjects are allowed to touch and press the sphere freely, and judge whether the sphere they interact with is soft, medium, or hard. There is no limit to the subject’s perception time. From the beginning of interaction to the completion of judgment is regarded as a perception process, and the subject’s judgment results and perception time are recorded. Each stiffness needs to be felt 10 times, and each subject needs to feel 30 times in total.

After the entire experiment, the subjects need to rate their level of agreement with the following four additional questions: “The device can enhance the authenticity of the interaction”, “I can clearly distinguish between three different stiffness levels of the sphere”, “During the use of the device, I am very satisfied with the interactive comfort” and “The visual 3D imaging synchronization is very good”. A 7-point scoring system is used, where 1 indicates “strongly disagree” and 7 indicates “strongly agree”.

### 5.3. Analysis of Results

**Figure 11:**
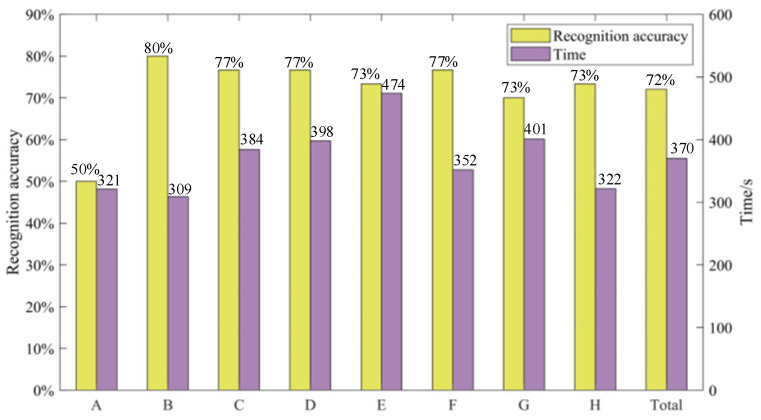
Perception accuracy and experiment completion time.

Figure 11 below shows the average perception accuracy of eight subjects and the time taken to complete the perception experiment. It can be seen that there is no obvious relationship between the time spent in the experiment and the accuracy.

**Figure 12:**
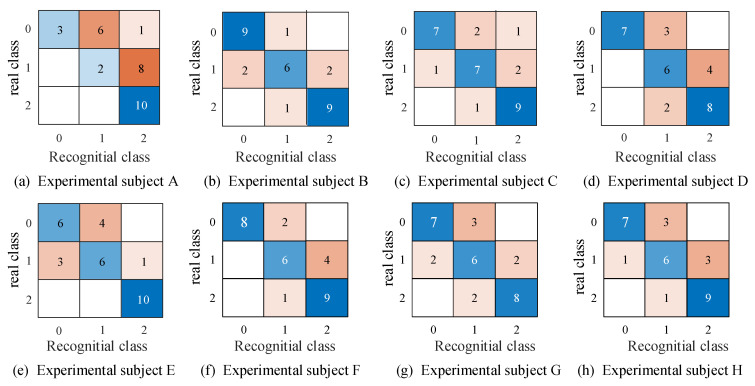
Confusion matrix of perception results. The (**a**–**h**) in the figure represent the perception results of subjects A–H respectively.

Figure 12 is the confusion matrix of subjects’ perception results, where 0, 1, and 2 represent spheres with soft, medium, and hard stiffnesses, respectively. The results shows that participants can accurately identify objects with a hard stiffness level, but struggle to identify objects with soft and medium stiffness levels. Additionally, users tend to misclassify objects with a soft stiffness level as medium, and objects with a medium stiffness level as hard. This suggests that users can distinguish between two extreme stiffness levels but struggle to differentiate between adjacent stiffness levels. Moreover, from Figure 12a, it can also be observed that subject A is prone to confusing soft and medium objects as medium and hard objects, respectively. Furthermore, different participants exhibit varying levels of perception regarding stiffness. Finally, the average identification rate of object stiffness achieved by this device while worn is 72%.

Table 3 shows the average results of the subjects’ recognition of the four sentences. From the experimental results, it is evident that the subjects all believe that the device can improve the authenticity of interaction. However, there are areas for improvement in terms of the comfort of wearing the device and its synchronization with visual cues.

## 6. Conclusions

This paper presents a wearable fingertip force feedback device for reproducing the stiffness of objects. The device is an independent system that separates the fingertip platform from the driving module using a combination of torsion springs and tendon drive, capable of providing a maximum feedback force of 4 N with a response speed of up to 10 N/s.

To fulfill the need for perceiving normal force feedback at the fingertip, a motor coupled with a screw slide mechanism is utilized. This mechanism converts the motor’s rotational motion into linear motion of the nut. The nut, in turn, pulls the fingertip platform via a torsion spring traction rope, thereby outputting normal force at the fingertip. The screw slide mechanism ensures precise coordination between the nut and the screw, effectively addressing issues related to swing and offset that can occur with direct drive motion. This enhancement improves the stability of the feedback force. Additionally, the use of torsion springs as an intermediate transmission element ensures a smoother output of the feedback force. An innovative fusion approach that integrates Leap Motion and posture sensors is employed to detect the position of the index fingertip within a three-dimensional interaction scene. Ultimately, the device’s capability to reproduce feedback force is validated through stiffness perception experiments. These experiments statistical values should be mentioned as on average 72 ± 5%, effectively demonstrating the device’s reliability.

In future work, further research is needed on issues such as the stability of the device to improve the feedback recognition effect of the device.

## Figures and Tables

**Table 1:** Parameters of the fingertip force feedback device.

Parameter	Index
*H*	200 mm
*LL*	25 mm
*LR*	30 mm
*X*	35 mm
*D_rα_*	0~24.8 mm
*θ_rα_*	0~1.446 rad
*D_sβ_*	0~20 mm
*β*	0~0.588 rad

**Table 2:** Response characteristics of different peak step signals.

Amplitude/N	Maximum Overshoot/%	Peak Time/ms	Response Time/ms
4.0	12.86	220	660
3.5	13.6	200	620
3.0	13.36	160	680
2.5	11.00	140	720
2.0	11.86	100	640
1.5	12.5	100	920
1.0	10.67	100	900
0.5	10.42	80	660

**Table 3:** The average ratings from the questionnaire survey.

Statements	Average Rating
S1: The device can enhance the authenticity of the interaction.	7
S2: I can clearly distinguish between three different stiffness levels of the sphere.	5.625
S3: During the use of the device, I am very satisfied with the interactive comfort.	5.75
S4: The visual 3D imaging synchronization is very good.	6.125

## Data Availability

The original contributions presented in the study are included in the article, further inquiries can be directed to the corresponding author.
